# Reconsidering the context in the relationship between material deprivation and self-rated health among older people in Italy

**DOI:** 10.1371/journal.pone.0345858

**Published:** 2026-04-20

**Authors:** Roberta Misuraca, Maria Carella

**Affiliations:** 1 Department of Sociology, Stockholm University Demography Unit, Stockholm, Sweden; 2 Department of Political Sciences, University of Bari “Aldo Moro”, Bari, Italy; University of Salamanca, SPAIN

## Abstract

Both personal characteristics and contextual factors shape an individual’s perception of their health. This is especially true among older people. Micro and macro data from the 2018 Italian Multipurpose Survey on Households and a two-step model have been used here to investigate the extent to which macro-level factors (at the regional level) explain variations in the nexus between material deprivation and self-rated health among older individuals in Italy, after considering micro characteristics. Our findings show that contextual variables, such as per capita GDP, tertiary education rates, and the availability of care home facilities, not only significantly moderate the effect of material deprivation on SRH but may paradoxically intensify its negative effects in wealthier or better-resourced regions. This counterintuitive pattern suggests that mechanisms of relative deprivation and social comparison are particularly salient in later life. These findings highlight the importance of context-sensitive, inclusive policy interventions that address both structural-economic disparities and the psychosocial realities of inequality to improve health outcomes for older populations.

## 1. Introduction

The global rise in the elderly population, with its resulting pressure on public finances, has led to an increased focus on improving health in later life. While economic factors alone do not fully explain the impact of different socio-contextual dimensions on individual health, researchers in the social sciences have looked beyond material factors to explore other intangible and less observable drivers of wellbeing [1].

Diverse methodological techniques have been applied to examine inequalities and the related concept of deprivation among various target groups [2–4]. Prior studies have often relied on income-based metrics because of their simplicity and accessibility [5,6]. However, these measures have limitations, as they focus on immediate financial status but fail to capture the multidimensional nature of deprivation, particularly among older adults, who are often *asset-rich but cash-poor* [7]. Measures of material deprivation may therefore provide an alternative by reflecting real-life conditions more accurately [8–12].

The elderly constitute a high-risk group due to their limited incomes, increased healthcare needs, and reduced labour market participation [13]. Additionally, poor housing conditions and health-related expenses place a further strain on their resources, making it harder to meet basic needs [14]. They may also face social isolation and vulnerability [15,16]. Collectively, these factors heighten their risk of material deprivation.

Prior studies have consistently shown that a deficient standard of living strongly predicts individual health outcomes [17–19]. Studies conducted in the European and Italian contexts show that various forms of material deprivation are closely linked to SRH [20,21], being influenced by a wide range of factors: not only do individual determinants directly impact individuals’ perceived health status, as socio-ecological considerations at the community level also shape such perceptions [22–24]. Sundry studies have shown that environmental determinants, including economic policies and social norms, might outweigh healthcare or lifestyle choices in influencing health [25,26].

While these studies examine the mediating role that the socioeconomic context plays in individuals’ health perceptions, they frequently overlook regional and individual characteristics as confounding variables in the link between material deprivation and SRH. Moreover, despite the evidence on the impact of contextual factors in European countries, the understanding of their contribution to SRH in Italian regions remains limited.

To bridge this gap, we leverage data from the 2018 Italian Multipurpose Survey on Households and adopt a micro–macro perspective, employing a two-step model to investigate how macro-level factors (at the regional level) explain variations in the material deprivation-SRH nexus among older individuals after considering micro characteristics (at the individual level). A two-step approach enables us to assess the moderating role that context plays in the estimated effect that material deprivation has on SRH, once individual-level variables have been considered.

The paper is structured as follows: Section 2 reviews the link between material deprivation and health, including individual and contextual influences. Section 3 details the data; Section 4, the methodology. Findings appear in Section 5, with robustness checks. Section 6 offers the discussion, limitations, and conclusions.

## 2. Literature review and theoretical background

Quality of life is no longer viewed solely through the lens of income or income-based poverty [27]; scholars have stressed the importance of incorporating measures of social exclusion and material deprivation [8,9], which extend beyond disposable income to provide a more comprehensive view of real living conditions [28]. The growing emphasis on the concept of social exclusion stems from its ability to capture real-life conditions [10–12], reflecting numerous interconnected dimensions of disadvantage and deprivation. While extensive research has explored the interplay between economic and social factors within a comprehensive definition of deprivation, there is no consensus on the definition of the term that encompasses both material and social needs [29]. This ongoing debate underscores the complexity of conceptualising deprivation in a way that addresses its multidimensional nature.

A commonly accepted definition [30,31], whereby deprivation is the inability to meet basic “*material*” needs (i.e., a proper diet, a warm home of a decent size and condition, possessing such tangible goods as a car, telephone, or personal computer, and enjoying domestic amenities such as an indoor flushing toilet). According to the Eurostat definition, material deprivation involves a progressive accumulation of limitations across numerous material and sociodemographic indicators that are generally “*considered by most people to be desirable or even necessary to experience an adequate quality of life*” [32]. This perspective is better suited to a comprehensive understanding of poverty in its broader meaning of material deprivation.

At the core of this study, extensive research has consistently reported a close connection between material deprivation and adverse health outcomes [16,27,33]. These studies show that deprived living standards are associated with poorer physical and mental health, higher levels of stress, and lower overall wellbeing [17,28]. Subjective measures of health, such as self-rated health (SRH), are especially well suited to capturing these processes, as they reflect both objective living conditions and individuals’ overall assessment of their health status [33,34].

Life-course theory and the Cumulative disadvantage perspective have long emphasized that the health consequences of socioeconomic disadvantage vary across the life course: exposure to hardship tends to accumulate over time, leading to widening health disparities in later life [35,36]. For older adults, material deprivation may therefore exert a particularly strong influence on health perceptions due to limited opportunities for recovery, reduced labour market participation, and increasing dependency on public and private support systems [37]. At the same time, some studies point to a partial “levelling” effect of age, whereby biological frailty becomes a dominant determinant of health outcomes irrespective of socioeconomic position [38]. However, rather than treating these perspectives as mutually exclusive, existing evidence suggests that individual vulnerability in later life may amplify or attenuate the health consequences of deprivation depending on broader social and contextual conditions [39].

### 2.1. A micro–macro perspective: Contextual moderation of deprivation-related health inequalities

Self-rated health is shaped by a wide range of factors operating at both the individual and contextual levels [40]. At the individual level, socioeconomic resources, living conditions, and personal characteristics such as age, education, household composition, and labour status play a central role in shaping health perceptions and vulnerability to poor health [16,33]. In this context, material deprivation can capture concrete constraints in daily life that directly affect individuals’ physical and psychological wellbeing.

At the same time, individual health outcomes are embedded within broader contextual environments.

A growing body of research adopts a micro–macro perspective, emphasizing that individual-level processes are embedded within regional socioeconomic, institutional, and infrastructural environments [41,42]. From this perspective, contextual factors do not merely exert direct effects on health but may also condition the strength of individual-level associations.

Empirical studies have shown that regional characteristics such as unemployment rates, income levels, educational attainment, and healthcare infrastructure are associated with variations in SRH and other health indicators [21,23,26]. Importantly, these factors may moderate how material deprivation translates into health outcomes by shaping access to resources, exposure to inequality, and prevailing standards of living.

This may appear particularly true in the case of Italy, where regional disparities in socioeconomic conditions are highly pronounced, often as strong as those observed between different national contexts. Regional contexts capture localized labor markets, welfare provision, healthcare infrastructure, and social environments that shape individuals’ living conditions and health beyond national averages. Taken together, these mechanisms suggest that material deprivation and self-rated health in later life cannot be fully understood by focusing solely on individual characteristics or national welfare arrangements. Rather, intra-country regional differences play a crucial role in shaping both the distribution of material hardship and its health consequences among older adults. For older adults in particular, differences in public service provision, healthcare availability, and social infrastructure can either mitigate or exacerbate the health consequences of material hardship [42]. As a result, identical levels of individual deprivation may lead to different health outcomes depending on the regional environment in which individuals are embedded [43].

Rather than treating contextual characteristics as competing explanations, we examine whether and to what extent regional socioeconomic conditions moderate the relationship between material deprivation and self-rated health among older adults.

### 2.2. The role of relative deprivation

The literature on deprivation emphasizes that disadvantage is experienced not only in absolute terms but also through social comparison processes that are inherently contextual. Individuals evaluate their living conditions relative to reference groups and prevailing social standards, rather than solely based on objective resource levels [44,45]. In this vein, perceptions of deprivation are shaped by judgments of fairness, legitimacy, and deservedness, which are strongly influenced by the surrounding socioeconomic environment [46].

A key implication is the reference to the relative deprivation theory, which states that upward social comparisons, particularly in contexts characterized by higher affluence or greater inequality, can generate psychosocial strain even among individuals facing similar levels of material hardship. Empirical evidence supports this view. For instance, previous studies show that individuals report lower levels of happiness during periods of greater income inequality, even after accounting for their own income [47]. Similarly, research documents higher mortality risks among low-income women residing in wealthier areas of California [48], while other evidence indicates increased levels of depression and psychological distress among disadvantaged families relocated to more affluent neighbourhoods [49]. Together, these studies suggest that contextual prosperity does not uniformly translate into better health outcomes and may, under certain conditions, exacerbate the health inequalities. These dynamics are particularly relevant for subjective health measures such as self-rated health, which capture individuals’ overall assessments of their health and wellbeing, including psychological and social dimensions that are not fully reflected by objective indicators [33,34]. Because SRH incorporates individuals’ interpretations of their circumstances, it is especially sensitive to relative evaluations and contextual benchmarks [50].

Furthermore, relative deprivation processes may be especially salient in later life, when individuals face reduced economic flexibility, declining health, and limited opportunities for social mobility. In this stage of the life course, material hardship experienced in more affluent or unequal contexts may be more readily internalized as a personal failure, reinforcing stress and negative health perceptions [51]. As a result, the health consequences of material deprivation may vary systematically across contexts, producing patterns that appear counterintuitive when regional environments are not explicitly considered.

Building on this theoretical perspective, the present study examines whether regional socioeconomic contexts condition the association between individual material deprivation and self-rated health among older adults.

**Fig 1:**
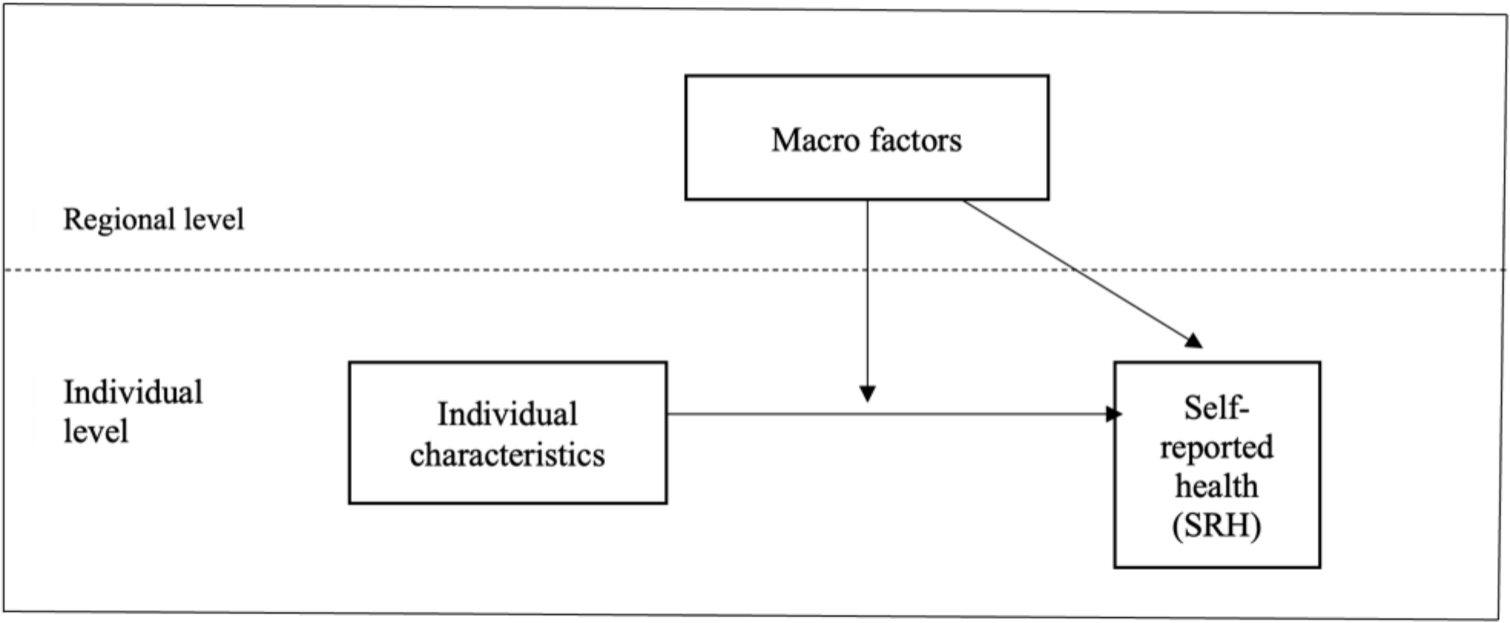
Conceptual framework of the study.

By focusing on cross-regional variation in the strength of this relationship, the analysis allows us to assess how contextual environments shape the health implications of material hardship, in line with insights from relative deprivation theory [46] and micro–macro approaches to inequality [41,42]. Fig 1 illustrates the conceptual framework guiding the analysis.

We therefore pose the following research questions:

1. Do macro-contextual factors at the regional level moderate the relationship between material deprivation and SRH among older people, and if so, to what extent?
2. What regional variations exist in the material deprivation-SRH relationship, and how do different contextual factors account for these differences?

## 3. Data

The empirical analysis is based on microdata from the 2018 Italian Multipurpose Survey on Households entitled Aspects of Daily Life, conducted by the Italian National Institute of Statistics (ISTAT), covering various aspects of living conditions. The primary objective is to improve our understanding of individual behaviours and daily challenges. Additionally, the survey provides critical insights into the work-life balance, interpersonal relationships, household dynamics, community engagement, political and social engagement, and healthy lifestyles. This enables us to explore the relationships between material deprivation and SRH across multiple dimensions of the participants’ lives.

Keeping in mind that contextual socioeconomic attributes are crucial variables, we also gathered macro-level data on various indicators reflecting different regional characteristics. These indicators were sourced from multiple databases compiled by ISTAT for the year 2018.

The data used in this study are publicly available through ISTAT and fully anonymised. As such, no specific informed consent was required.

After selecting individuals aged 55 and over, our sample consisted of 15,713 participants. We chose to focus on this cohort to explore how varying degrees of material deprivation affect them, segmenting them by age (55–64 and 65 and over) and by characteristics commonly associated with each one accordingly.

### 3.1. Dependent variable

Our main variable of interest is Self-Rated Health (SRH). We gathered information on the respondents’ perception of their state of health over the previous 12 months. We posed the following question: *How is your health in general?* SRH was evaluated using a 5-point Likert scale, from 1 (indicating *very good*) to 5 (indicating *very bad*). We chose SRH because it provides valuable information on individuals’ general health and constitutes a good indicator of their perceived health [52], even considering the subjective nature of the material deprivation variable. A dummy variable was created and assigned a value equal to 1 for poor health perception (ranging from 5 to 3 on the ordinal scale variable) and 0 otherwise. Given that the SRH measure in our survey was ordinal, an ordinal variable would have been the conventional choice. However, given the subjective nature of the scores and the characteristics of both SRH and individual material deprivation, a binary choice was more appropriate, as it allowed for an easier interpretation of the results. Nonetheless, an ordinal dependent variable is used in the Appendix for additional analysis (see Table A4).

### 3.2. Individual-level variables

Our main explanatory variable is a measure of material deprivation. The methodological framework employed in this study is based on the premise that material deprivation is a multifaceted concept encompassing various aspects of daily life, not exclusively linked to an individual’s financial status. We therefore gathered data on the material and sociodemographic indicators of deprivation assessed by a set of items that includes being able to afford meat, chicken, and fish; maintaining a sufficiently warm home; having certain durable goods such as a car, personal computer, telephone, and washing machine; and the availability of housing amenities such as an indoor flushing toilet. Furthermore, we examined aspects such as the size and condition of the home and local environmental factors, including pollution, crime, violence, and noise, as well as people’s ability to cover essential expenses, such as housing costs. The scale for measuring food and household conditions ranges from 1 to 3 (more than twice a week/once a week/rarely, or never), while dummy variables are used for other deprivation items. Table 1 lists the items included in the index and their respective min-max values.

**Table 1:** List of Items used to build Material Deprivation Index.

Category	Deprivation Items	Measurement Scale
Food deprivation	Ability to afford meat, chicken, or fish	1 = More than twice a week 2 = Once a week 3 = Rarely or never
Housing conditions	Ability to maintain a sufficiently warm home	Dummy (0 = No deprivation, 1 = Deprived)
Durable goods	Ownership of car, personal computer, telephone, washing machine	Dummy (0 = Possess, 1 = Do not possess)
Housing amenities	Availability of an indoor flushing toilet	Dummy (0 = Available, 1 = Not available)
Home condition	Size and condition of the home	Dummy (0 = Adequate, 1 = Inadequate)
Local environment	Exposure to pollution, crime, violence, noise	Dummy (0 = No issues, 1 = Issues present)
Financial strain	Ability to cover essential expenses (e.g., housing costs)	Dummy (0 = Can afford, 1 = Cannot afford)

Source: ISTAT, 2018.

Even considering the various methods in the literature, operationalising the construction of the index of material deprivation remains a difficult exercise due to the choice of items, their volatility, and the validity of the scale, which could be critical [53].

Given the multidimensional nature of material deprivation and the heterogeneous format of the items, after gathering data on the main deprivation indicators and their frequency, we conducted a Latent Class Analysis (LCA) to construct a measure of high material deprivation based on different combinations of indicator frequency and variety. We cluster individuals reflecting comparable deprivation patterns in terms of the intensity of their hardship and the variety of deprivation indicators and derive a measure of the individual conditional marginal probability of being highly materially deprived. As a result, our explanatory variable captures the likelihood of experiencing the highest levels of material deprivation concerning various deprivation indicators at the individual level (see Appendix for model specification). For the main analysis, we transformed this classification into a binary indicator of high material deprivation, assuming a value of 1 for maximum perceived discomfort and 0 otherwise. Cronbach’s alpha for the ten deprivation items is modest (α ≈ 0.15), which is typical for multidimensional material deprivation constructs combining heterogeneous conditions [9,10]. Because the items capture distinct but related forms of hardship rather than a single latent continuum, low internal consistency is theoretically expected. This further justifies the use of LCA to summarise deprivation patterns.

As a robustness check (Table A3 in S1 Appendix), we also use the continuous posterior probability of belonging to a highly deprived profile, which preserves within-class variability and reflects the individual likelihood of experiencing high deprivation.

### 3.3. Regional-level variables

Per capita GDP has been used to reflect regional competitiveness and economic security, specifically for Purchasing Power Standards, to explore whether the material deprivation-SRH nexus among older adults is influenced by the level of regional economic performance. We also consider a measure of income inequality by including the poverty index. This index is calculated as the percentage of households in relative poverty by region. We refer to the Poverty Index by ISTAT, calculated using a poverty threshold that varies by household characteristics and geographical location. Specifically, a household is in poverty if its total monthly consumption expenditure is below the absolute poverty threshold, which is the minimum amount required to purchase a basket of essential goods and services, such as food (caloric intake needed for adequate nutrition), housing (rent, utilities, maintenance), clothing and personal care, health care (medicine, doctor visits), education and communication. These costs are determined separately for different types of households and geographical areas, such as region (Northern, Central, and Southern Italy) and urbanization level (metropolitan area vs. small towns). The threshold also varies by household size and composition (e.g., single adults, couples, families with children, elderly individuals).

This measure provides a social indicator of the level of economic hardship in the surrounding environment. Some studies have shown that income inequality and a widespread perception of poverty negatively impact individual satisfaction from different points of view, including SRH [54,55].

We also include a further measure of local economic stress. The unemployment rate is measured as the number of individuals seeking employment within the resident population for the year under analysis.

Regarding the environmental domain, we consider the average level of air pollution for each region (CO2 Emissions). Specifically, while residing in urban areas may enhance economic and social opportunities, it can also lead to increased stress [56]. Previous studies [57] have indeed noted that high levels of industrialisation, coupled with the extensive use of private vehicles, lead to increased exposure to air pollution among residents. This exposure negatively affects both life satisfaction and health outcomes [58].

We also consider the influence of education, specifically by controlling for the effect of the regional tertiary education rate, defined as the proportion of individuals aged 25–64 who have attained tertiary education within the resident population of the same age group. We use this measure as a proxy for the level of socioeconomic development and the potential for innovation and growth in the region [59], as well as an indicator of human capital and skill levels. Previous studies on subjective wellbeing among older people suggest that higher levels of education are often linked to greater health awareness and healthier lifestyles, which could reflect regional health trends [60–62].

Finally, we include several indicators of healthcare accessibility: the number of hospital beds, the number of elderly care facilities, and the number of beds in care facilities. These measures reflect the healthcare system’s capacity to address the care needs of older adults. Together, they also serve as proxies for the overall quality and accessibility of services available to the aging population within the region [63], as well as the regional priorities and resource allocation regarding health and elderly care services.

## 4. Methodology

A two-step approach is applied to explore whether and to what extent the socioeconomic characteristics of the context moderate the material deprivation-SRH relationship. The core notion is that individuals’ perceived health is influenced not only by personal traits but also by their environment. Objective macro-level factors may therefore significantly shape individual perceptions of health and overall wellbeing. This is especially true for older adults, where macro-level factors can interact more strongly with individual characteristics to explain variations in the material deprivation-SRH nexus.

We adopt a two-step approach for two main reasons: a multistep process could be advantageous in the presence of hierarchically structured data, where individual data (lower level) are nested within regional units (higher level). This approach allows estimating the moderating effect of higher-level data on the overall impact, as well as the separate effects of individual variables on the main dependent variable, namely, SRH [58]. On the other hand, in a scenario (as in our case) in which large samples of individuals are within a limited number of units (regions), a two-step approach should enable us to assess the degree of variability at each level of analysis. This provides us with a more comprehensive understanding of the interplay between contextual and individual factors in shaping individuals’ experiences and preferences, thereby fully explaining how a combination of different levels influences the relationship between material deprivation and SRH.

Following the existing literature [58], our first step is based on the following formula:


$${SRH}_{i,r}=\;\alpha_{r}+\beta_{\text{1}}{DI}_{i,r}+{\beta^{\prime }}_{\text{2}}X_{i,r}+u_{i,r}$$

Where *SRH* is the self-rated health of individual *i* in region *r,* that assumes a value of one in the case of poor SRH, and 0 otherwise. Our explanatory variable is *DI*, which refers to the deprivation index for individual *i* in region *r*. The index assumes a value of 1 in the case of maximum perceived hardship, and 0 otherwise. The parameter β1 represents the relationship between the deprivation index and an individual’s SRH. A positive and statistically significant coefficient indicates that a higher degree of social and economic hardship is associated with a more negative perception of health status.

The vector 𝑋′ comprises individual-level observable characteristics commonly recognised in the literature as significantly influencing *SRH.* It includes variables such as gender, age, partnership status (married/cohabiting, separated/divorced, widow/er, or single), educational attainment (upper-secondary and tertiary levels, or little education), living arrangements (living alone, with family members, with distant relatives, or cohabiting with housemates or caregivers), and labour status (currently employed, retired, or unemployed). Different income sources were also considered (employment income, self-employment income; pension, allowance, property income, family maintenance). Considering these control variables, we can account for relevant individual traits that might have influenced material deprivation patterns and SRH outcomes. Finally, $u_{ir}$ is the error term. The descriptive statistics of the variables are displayed in Table A1 in S1 Appendix.

In this first step of the analysis, the same model was estimated separately for each region using a Linear Probability Model (LPM) to assess the relationship between material deprivation and SRH. The use of OLS ensures comparability of coefficients across regions, avoids the scale identification problems inherent to nonlinear models [64], in this direction allowing a coherent application of the Estimated Dependent Variable (EDV) framework [58]. In the second step, we investigate whether regional-level contextual variables moderate this relationship. Specifically, the estimated regional coefficients of *DI* on SRH ($\hat{\beta }$), obtained from the first step) were regressed on the region-specific variables $Z_{r}$, using the feasible generalised least squares model (FGLS) to correct for estimation errors in the estimated coefficients from the first step [64], aligning with prior applications in regional research [58]. FGLS accounts for the heteroskedasticity arising from using first stage estimated coefficients as dependent variables, weighting observations according to their relevance. The related formula is the following:


$$\hat{\beta }\text{1},r=\;\gamma_{\text{0}}+\;\gamma^{\prime }\;Z_{r}+\;\eta_{r}$$

## 5. Results

### 5.1. Cross-regional variations

**Fig 2:**
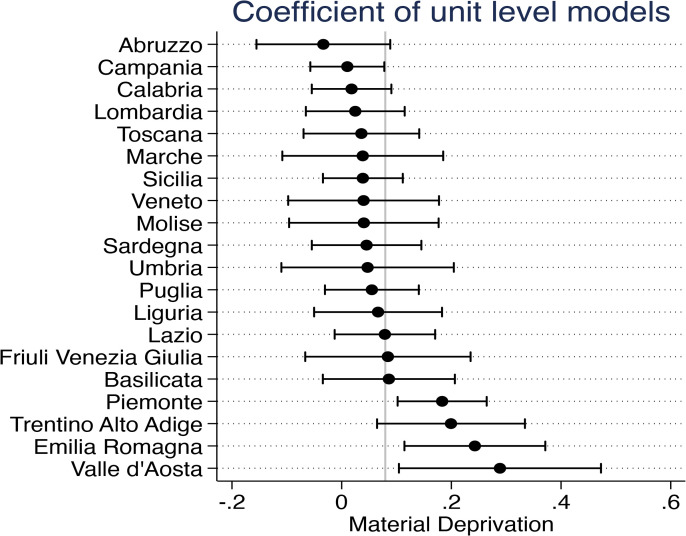
The marginal effect of material deprivation on SRH. Note: the figure shows the estimated 95% confidence intervals of the marginal effects of the material deprivation dummy variable on SRH at individual level in each region, adjusted for gender, age, partnership status (married/cohabiting, separated/divorced, widow/er, or single), educational attainment (upper-secondary and tertiary levels, or little education), living arrangements (living alone, with family members, with distant relatives or sharing with housemates or caregivers), labour status (currently employed, retired, or unemployed), and different income sources. The reference population consists of individuals aged 65 and older. Points represent region-level coefficients from first-stage regressions, while horizontal lines indicate 95% confidence intervals. The vertical line denotes the null effect. Estimates are obtained from region-specific linear probability models controlling for individual-level characteristics.

Fig 2 illustrates the estimated coefficients from region-specific models assessing the relationship between material deprivation and SRH across Italian regions. Each dot represents the marginal effect of deprivation on the likelihood of reporting poor SRH, controlling for individual-level characteristics. While only a subset of regional coefficients is statistically significant at conventional levels, Fig 2 reveals substantial heterogeneity in the magnitude and direction of the deprivation–SRH association across regions. Our interpretation, therefore, focuses on regional variation patterns rather than on statistical significance alone, which is expected to be limited given the small number of higher-level units.

Overall, the results show a positive association across most regions, suggesting that individuals experiencing higher levels of deprivation are more likely to report poorer health. However, the strength and significance of this relationship vary considerably by region. For example, Valle d’Aosta, Emilia Romagna, and Trentino Alto Adige (regions located in the North of Italy) exhibit relatively strong and significant effects, whereas regions such as Abruzzo and Campania (in Southern Italy) display weaker or no association (closer to zero).

These differences may reflect underlying contextual heterogeneity due to a combination of socioeconomic, cultural, and structural factors, such as the role of social comparisons and higher normative expectations regarding living standards and health or, for instance, differences in social structures, service provision, and informal support systems that may mediate this relationship. While these interpretations remain speculative, they help contextualise the observed heterogeneity and motivate the subsequent analysis of regional-level moderators.

### 5.2. Results of the moderator effect of regional-level variables

Table 2 presents the results of the second-step analysis, which explores how regional-level contextual variables are associated with the relationship between material deprivation and SRH among older adults.

**Table 2:** Regressions FGLS – Moderating effects of regional-level variables on the relationship between material deprivation and SRH.

VARIABLES	(1)	(2)	(3)	(4)	(5)	(6)	(7)	(8)
Unemployment rate	−0.00784**							
	(0.00326)							
Per capita GDP		6.411***						
		(2.007)						
CO2 emissions			0.103***					
			(0.0224)					
Poverty index				−0.00564**				
				(0.00223)				
Tertiary education rate					0.00808**			
					(0.00362)			
Number of beds in hospital facilities						0.196***		
						(0.0443)		
Elderly care facilities							0.00585***	
							(0.00104)	
Number of beds in care facilities								0.000176***
								(4.63e-05)
Constant	0.169***	−0.0974	−0.131**	0.154***	−0.140	−0.526***	−0.0656**	−0.0495
	(0.0397)	(0.0586)	(0.0485)	(0.0326)	(0.101)	(0.139)	(0.0289)	(0.0377)
Observations	20	20	20	20	20	20	20	20
R-squared	0.244	0.362	0.539	0.263	0.217	0.520	0.637	0.447

Standard errors in brackets; *** p < 0.01, ** p < 0.05, * p < 0.010. The reference population consists of individuals aged 65 and over. Each column (1–8) represents a separate regression model including one regional-level variable at a time. The dependent variable is ($\hat{\beta }$), the estimated effect of *DI* on *SRH.*

From a general point of view, the effect of material deprivation on poor SRH is stronger in regions with higher per capita GDP (6.411), greater tertiary education attainment (0.00808), and more developed healthcare infrastructure, as indicated by the number of hospitals (0.196). These results may appear counterintuitive at first glance, as such regions are generally more affluent and better equipped to support public health. Conversely, the effect of material deprivation on SRH appears weaker in regions with higher unemployment (−0.00784) and poverty rates. In such contexts, hardship may be more widespread and socially normalised, reducing the relative psychological burden of individual deprivation.

Interestingly, environmental factors such as CO₂ emissions also appear to play a moderating role, with higher emissions associated with a stronger link between deprivation and poor SRH. Urban residents facing deprivation might therefore experience a more jarring contrast between their needs and the opportunities available around them, especially if services are present but remain inaccessible due to financial barriers.

**Fig 3:**
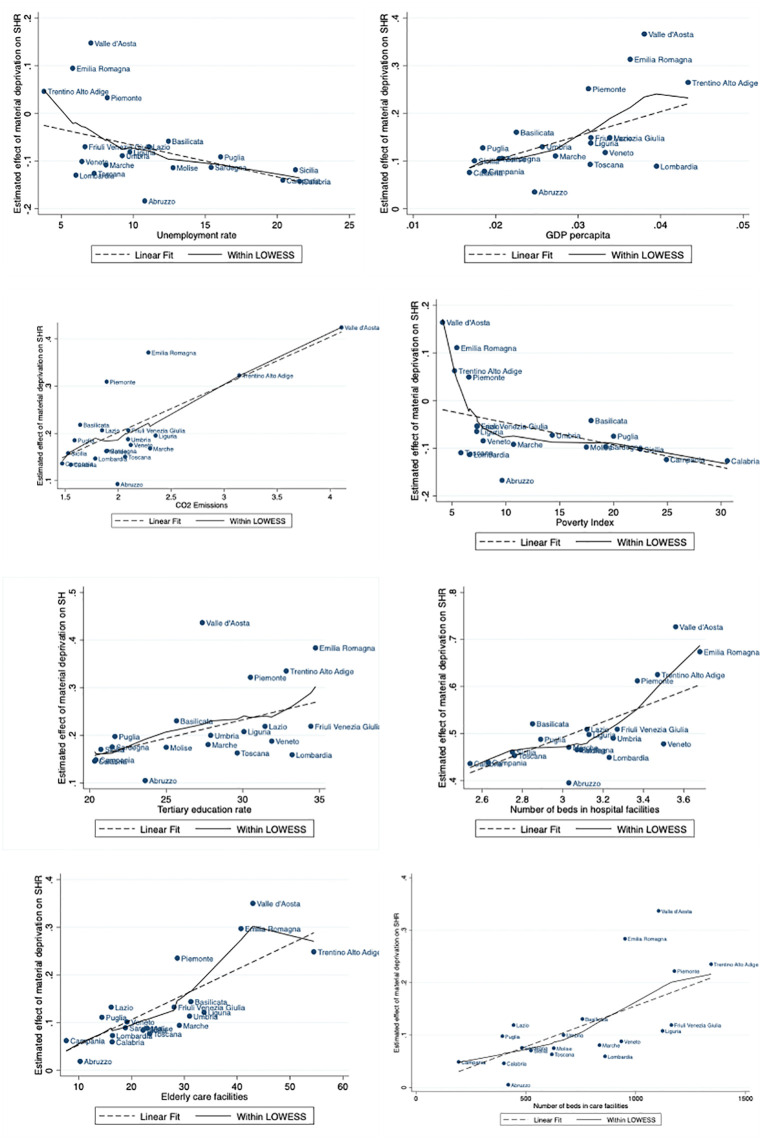
Estimated impact of material deprivation on SRH across regions for different indicators. Note: Figures display the regions’ point estimates, regressing the marginal effect of each variable on the estimated impact of material deprivation on SRH. The reference population consists of individuals aged 65 and over. Material deprivation profile derived using the LCA approach and subsequently converted into a binary indicator of high material deprivation, coded as 1 for maximum perceived discomfort and 0 otherwise.

Fig 3 visually supports the regression findings presented in Table 2 by showing the relationship between the estimated regional effect of material deprivation on SRH and various regional-level contextual variables. Each point represents a specific region. For instance, a clear negative slope is visible in the plots for unemployment rate and poverty index, confirming that in regions with higher unemployment or poverty, the impact of deprivation on SRH is weaker, likely due to social normalisation of hardship or stronger informal safety nets. Conversely, positive slopes in the graphs for GDP per capita, CO₂ emissions, and healthcare infrastructure (such as hospital beds and elderly care facilities) indicate that in wealthier and more developed regions, deprivation has a stronger negative effect on perceived health.

Finally, looking at the population aged 55–64, our findings reveal weaker coefficients (especially for per capita GDP, CO2 emissions and healthcare accessibility) (Table A2 in S1 Appendix). The differences between the two age groups can be attributed to variations in age-related vulnerabilities, healthcare needs, and social conditions.

### 5.3. Robustness checks

We further validate our findings by exploring the potential role of regional-level variables on the relationship between high material deprivation and SRH using different approaches. First, we replicate our main two-step model using the continuous LCA posterior probability instead of the binary indicator (Table A3 in S1 Appendix). This approach allows us to capture within-class variation in deprivation intensity and test whether the association with SRH increases gradually rather than only above a threshold. This complements the binary indicator, which identifies the most materially deprived group but does not exploit the full richness of the latent class model.

Then, we treat SRH as ordinal to better capture marginal differences in health perception among older individuals (Table A4 in S1 Appendix) and finally, as a further check, we estimated multilevel logistic regression models with random intercepts at the regional level and cross-level interactions between individual material deprivation and regional contextual characteristics (Table A5 in S1 Appendix).

The intraclass correlation coefficient (ICC) from a null random-intercept model was 0.016 (SE = 0.006), indicating that approximately 1.6% of the variance in SRH lies between regions. This non-zero ICC justifies the use of multilevel modelling or, in general, the inclusion of a regional-level. Furthermore, the different values of the constant by region in Table A5 indicate persistent, non-negligible heterogeneity in SRH across regions even after adjusting for individual and contextual characteristics.

**Fig 4:**
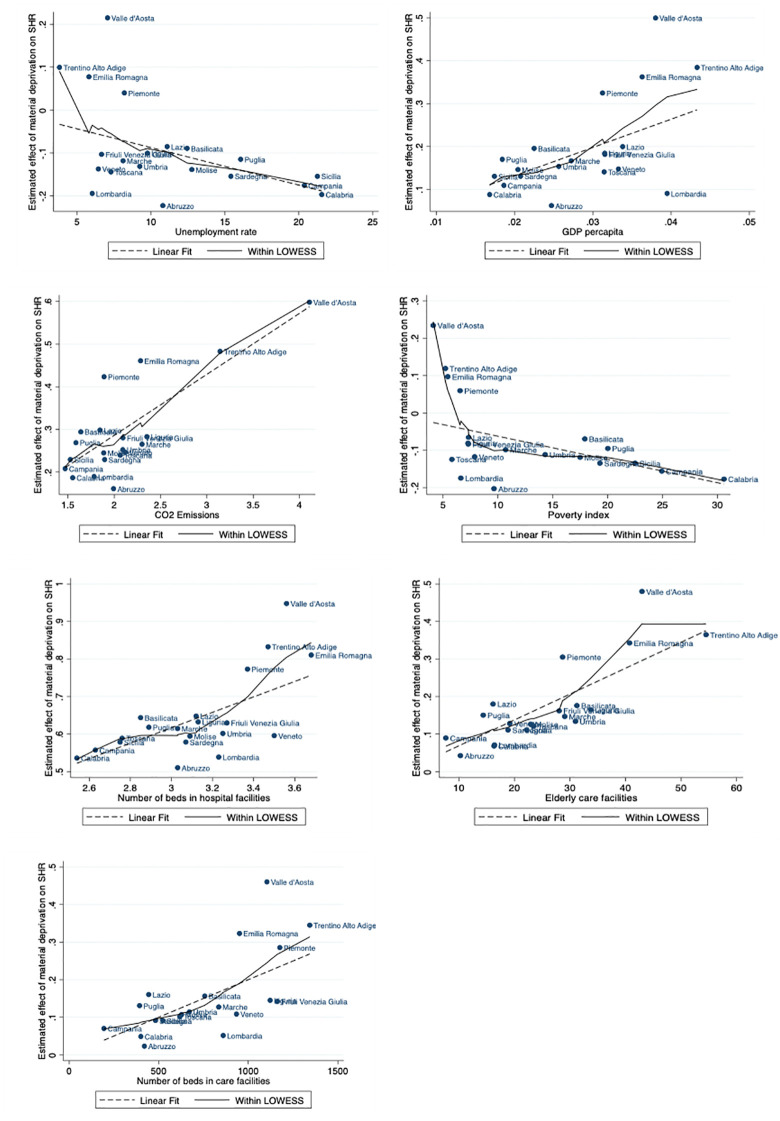
Estimated impact of material deprivation on SRH across regions for different regional-level indicators. Note: Figures display the regions’ point estimates, regressing the marginal effect of each regional-level variable on the estimated impact of material deprivation profile on SRH. The reference population are individuals aged 65 and older. Material deprivation profile derived using the LCA approach, expressed as a continuous posterior probability of belonging to the highly deprived profile.

From a general perspective, the coefficients in Table A3 confirm our results, further validating our main findings on the role of regional-level variables in shaping the impact of material deprivation on SRH among older adults. Interestingly, the overall results reveal stronger and more nuanced effects. For instance, the coefficients for per capita GDP, CO2 emissions and number of beds in hospital facilities are higher in the LCA model than in the dummy one, providing more detailed information about the incremental magnitude of change and further confirming the consistency of our findings, which are further supported by the results presented in Fig 4, where the slope and direction of the relationship align with and validate our conclusion.

Secondly, in Table A4, we use an ordinal SRH variable, evaluated using a 5-point Likert scale, from 1 (indicating *very good*) to 5 (indicating *very bad*) over the past 12 months. The fact that the results retain both their magnitude and direction further confirms the robustness of our findings.

Finally, in Table A5, across all regional characteristics considered, the estimated effect of material deprivation on poor SRH remains significant and of similar magnitude to the main model. Moreover, in most cases, the interaction terms indicate that the association between deprivation and SRH becomes stronger in socioeconomically disadvantaged or environmentally stressed regions, suggesting a contextual amplification of individual hardship. Notably, the coefficients for high material deprivation are slightly larger than those obtained in the two-step approach, indicating that the multilevel specification captures an even clearer relationship between deprivation and health.

This consistency across different analyses underscores the reliability of the observed relationships and strengthens the validity of our results.

## 6. Discussion and conclusions

SRH is shaped by a broad spectrum of factors operating at multiple levels [40]. While individual determinants directly affect perceived health status, local socio-ecological factors indirectly influence individuals’ perceptions of their health, and this dynamic appears to be especially pronounced among older adults experiencing material deprivation [43].

This study aims to investigate whether and to what extent regional socioeconomic contexts shape the relationship between material deprivation and self-rated health among older adults in Italy. The results provide clear evidence of substantial cross-regional heterogeneity in the strength of this association, even after accounting for a comprehensive set of individual-level characteristics. In other words, identical levels of individual material deprivation are associated with different health perceptions depending on the regional environment in which older individuals are embedded.

Consistent with previous literature [65,66], we found that material deprivation has a detrimental health effect in our specific cohort. Beyond this well-established relationship, however, the analysis highlights that macro-contextual factors, such as regional economic conditions, educational composition, environmental stressors, and healthcare infrastructure, moderate the deprivation–SRH nexus in meaningful ways. These results align with micro–macro perspectives emphasizing that individual outcomes are embedded within broader socioeconomic and institutional environments [41,42].

A particularly striking and counterintuitive finding concerns more affluent and socioeconomically developed regions. In regions characterized by higher per capita GDP, higher tertiary education rates, and more extensive healthcare infrastructure, material deprivation is associated with a stronger negative effect on self-rated health among older adults. This result does not imply that wealthier regions generate worse health outcomes overall; rather, it suggests that the health consequences of individual deprivation may be amplified in contexts where higher standards of living, greater service availability, and stronger normative expectations prevail. One possible interpretation of this pattern draws on the literature on relative deprivation, which emphasizes that disadvantage is experienced not only in absolute terms but also through social comparison processes that are inherently contextual [44,46]. In more affluent or highly educated regions, older adults facing material hardship may evaluate their living conditions against higher reference standards, potentially intensifying the perceived health impact of deprivation. This relative disadvantage can intensify feelings of exclusion, stress, and social invisibility, all of which have been linked to poorer health outcomes. Subjective health measures such as SRH are particularly sensitive to these contextual benchmarks, as they incorporate individuals’ overall assessments of physical, psychological, and social wellbeing [33,34].

Alternative explanations may also account for the observed patterns. First, compositional differences across regions may play a role: deprived older adults residing in wealthier regions may differ systematically from their counterparts elsewhere in terms of health history or family resources. Second, reporting heterogeneity cannot be ruled out. Higher levels of health literacy and awareness in more developed regions may lead individuals to report health problems more readily. Third, unobserved contextual factors, such as differences in regional welfare governance or the presence of informal support systems, may confound the association between deprivation and SRH.

The moderating role of healthcare infrastructure further illustrates these complexities. From a general perspective, knowing that healthcare resources are available creates a sense of security, especially among older adults needing frequent or urgent care [67]. This sense of security is particularly important for individuals already experiencing the challenges of material deprivation [68]. However, our findings seem to suggest that regions with greater healthcare accessibility are also those where material deprivation has a stronger negative impact on SRH. Rather than indicating a detrimental role of healthcare provision per se, this finding may suggest that the presence of healthcare resources may be insufficient to mitigate subjective health inequalities if access remains uneven or if services are perceived as difficult to navigate. It is also possible that regions with more developed healthcare systems attract or retain individuals with poorer health, or that more intensive service provision increases diagnosis and awareness of health problems, thereby affecting self-rated health.

In contrast, regions with higher unemployment and poverty rates exhibit a weaker association between material deprivation and SRH. One possible interpretation is that in contexts where hardship is more widespread, deprivation may be perceived as a shared condition rather than an individual failure, reducing its psychological salience [46]. Moreover, in some Southern regions, stronger family ties and informal support networks may partially compensate for material hardship and institutional shortcomings, thereby buffering their perceived impact on health, consistent with prior research on social normalization and informal support [43,48].

Taken together, these findings underscore the importance of moving beyond one-size-fits-all interpretations of health inequalities in later life. Psychosocial processes emphasized by relative deprivation theory [46,50,51] offer one possible explanation for why subjective health may deteriorate even in seemingly advantaged contexts.

At the same time, the results clearly indicate that material deprivation does not translate into poor self-rated health uniformly across contexts; rather, its impact is shaped by the surrounding socioeconomic, institutional, and environmental conditions. This highlights the importance of explicitly incorporating contextual moderators when studying health inequalities among older populations.

From a policy perspective, these findings indicate that reducing health inequalities among older adults requires context-sensitive interventions rather than uniform solutions. In ageing societies such as Italy, policies should go beyond purely structural improvements and address social inequality, accessibility, and subjective wellbeing, particularly for older individuals facing material hardship. In more affluent regions, efforts should focus on improving equitable access to existing services and reducing economic and administrative barriers, while in less affluent regions strengthening social support mechanisms and addressing broader socioeconomic vulnerabilities may be especially important. Overall, the results highlight the need to integrate individual-level interventions (e.g., targeted financial support for the materially deprived) with place-based and regional strategies that take local socioeconomic conditions into account, while also considering age-specific vulnerabilities when designing policies to promote health in later life.

This work is not without limitations. It is based on a cross-sectional dataset, which may raise concerns regarding the causality of the findings. To address these potential biases, the study’s empirical estimations were enriched by a comprehensive set of robustness checks. These tests were designed to assess the consistency of our results across different model specifications, alternative measures of key variables, and sample restrictions.

In conclusion, this study contributes to the literature by demonstrating that the relationship between material deprivation and self-rated health among older adults is context dependent. By highlighting the multidimensional nature of these effects, the findings draw attention to the critical role that local contextual factors, specifically the socioeconomic conditions of the surrounding environment, play in shaping the impact of material deprivation on SRH among the elderly, emphasizing the need for region-specific and multidimensional policy approaches that address not only material conditions but also access, inclusion, and subjective wellbeing in ageing societies such as Italy.

## Supporting information

S1 Appendix Supplementary analyses and robustness checks. This appendix reports additional descriptive statistics (Table A1 in S1 Appendix), second-step FGLS estimates for the population aged 55–64 (Table A2 in S1 Appendix), robustness checks using the continuous latent class posterior probability of material deprivation derived from the Latent Class Analysis (Table A3 in S1 Appendix), alternative model specifications treating self-rated health as an ordinal outcome (Table A4 in S1 Appendix), and multilevel logistic models with random intercepts and cross-level interactions between individual material deprivation and regional contextual factors (Table A5 in S1 Appendix). It also provides methodological details on the construction of the material deprivation indicator based on Latent Class Analysis.
(DOCX)

## References

[1] Alkire S, Foster J. Counting and multidimensional poverty measurement. Journal of Public Economics. 2011;95(7–8):476–87. doi: 10.1016/j.jpubeco.2010.11.006

[2] Navarro C, Ayala L. Multidimensional housing deprivation indices with application to Spain. Applied Economics. 2008;40(5):597–611. doi: 10.1080/00036840600722323

[3] Nolan B, Whelan CT. On the multidimensionality of poverty and social exclusion. Oxford Bulletin of Economics and Statistics. 2007;69(2):229–56.

[4] Panek T. Multidimensional approach to poverty measurement. Stat Transit. 2010;11(2):201–25.

[5] Fisher J, Johnson D, Smeeding T. Inequality of income and consumption in the US: Measuring the trends in inequality from 1984 to 2004. Review of Income and Wealth. 2009;55(3):505–30.

[6] Ringen S. Direct and indirect measures of poverty. J Soc Pol. 1988;17(3):351–65. doi: 10.1017/s0047279400016858

[7] Sullivan JX, Turner L, Danziger S. The relationship between income and material hardship. J Policy Anal Manage. 2007;27(1):63–81. doi: 10.1002/pam.20307

[8] Andress H-J, Lohmann H. The working poor in Europe: Employment, poverty and globalization. Edward Elgar Publishing. 2001.

[9] Boarini R, d’Ercole MM. Measures of material deprivation in OECD countries. 2006.

[10] Whelan CT. Understanding the mismatch between income poverty and deprivation: A dynamic comparative analysis. European Sociological Review. 2004;20(4):287–302. doi: 10.1093/esr/jch029

[11] Nolan B, Whelan CT. Using non‐monetary deprivation indicators to analyze poverty and social exclusion: Lessons from Europe?. J Policy Anal Manage. 2010;29(2):305–25. doi: 10.1002/pam.20493

[12] Guio AC. What Can Be Learned From Deprivation Indicators In Europe?. 2009.

[13] Levy H. Income, poverty and material deprivation in old age: An international perspective. Börsch-Supan A, Kneip T. Ageing in Europe – Supporting policies for an inclusive society. De Gruyter. 2016. 201–22.

[14] Bartlett H, Warburton J, Lui C-W, Peach L, Carroll M. Preventing social isolation in later life: Findings and insights from a pilot Queensland intervention study. Ageing and Society. 2012;33(7):1167–89. doi: 10.1017/s0144686x12000463

[15] Tang VFY, Chou KL. An exploratory study on material deprivation and loneliness among older adults in Hong Kong. BMC Geriatr. 2024;24(1):400. doi: 10.1186/s12877-024-05013-1 38711009 PMC11071256

[16] Terraneo M. The effect of material and social deprivation on well-being of elderly in Europe. Int J Health Serv. 2021;51(2):167–81. doi: 10.1177/0020731420981856 33342332

[17] Foulds J, Wells JE, Mulder R. The association between material living standard and psychological distress: Results from a New Zealand population survey. Int J Soc Psychiatry. 2014;60(8):766–71. doi: 10.1177/0020764014521394 24553670

[18] Groffen DAI, Bosma H, van den Akker M, Kempen GIJM, van Eijk JTM. Material deprivation and health-related dysfunction in older Dutch people: Findings from the SMILE study. Eur J Public Health. 2008;18(3):258–63. doi: 10.1093/eurpub/ckm119 18160391

[19] Oakes JM, Rossi PH. The measurement of SES in health research: Current practice and steps toward a new approach. Social Science & Medicine. 2003;56(4):769–84. 12560010 10.1016/s0277-9536(02)00073-4

[20] Halleröd B, Larsson D. Poverty, welfare problems and social exclusion. International Journal of Social Welfare. 2008;17(1):15–25.

[21] Franzini L, Giannoni M. Determinants of health disparities between Italian regions. BMC Public Health. 2010;10:296. doi: 10.1186/1471-2458-10-296 20515482 PMC2902435

[22] Pampel FC, Krueger PM, Denney JT. Socioeconomic disparities in health behaviors. Annual Review of Sociology. 2010;36:349–70. 10.1146/annurev.soc.012809.102529 PMC3169799 21909182

[23] Costa G, Caiazzo A, Marinacci C, Spadea T. Individual and contextual determinants of inequalities in health: The Italian case. The political and social contexts of health. Routledge. 2020. 115–46. 10.2190/AM8R-K0DY-F7PM-3RNP 14758854

[24] Short SE, Mollborn S. Social determinants and health behaviors: Conceptual frames and empirical advances. Curr Opin Psychol. 2015;5:78–84. doi: 10.1016/j.copsyc.2015.05.002 26213711 PMC4511598

[25] World Health Organization WHO. Social determinants of health equity: The evidence base for policy action on social determinants of health. Geneva: WHO Press. 2011.

[26] Rentería E, Zueras P. Macro-level factors explaining inequalities in expected years lived free of and with chronic conditions across Spanish regions and over time (2006-2019). SSM Popul Health. 2022;19:101152. doi: 10.1016/j.ssmph.2022.101152 35865801 PMC9293933

[27] Myck M, Najsztub M, Oczkowska M. Implications of social and material deprivation for changes in health of older people. J Aging Health. 2020;32(5–6):371–83. doi: 10.1177/0898264319826417 30694097

[28] Pfoertner T-K, Andress H-J, Janssen C. Income or living standard and health in Germany: different ways of measurement of relative poverty with regard to self-rated health. Int J Public Health. 2011;56(4):373–84. doi: 10.1007/s00038-010-0154-3 20495993

[29] Pirani E. Poverty and social exclusion in Europe: Differences and similarities across regions in recent years. 2013.

[30] Jehoel-Gijsbers G, Vrooman C. Social exclusion of the elderly: A comparative study of EU member states. 2008.

[31] Myck M, Najsztub M, Oczkowska M. Measuring social deprivation and social exclusion. Ageing in Europe—Supporting policies for an inclusive society. Berlin: De Gruyter. 2015. 67–78.

[32] Eurostat. Living Conditions in Europe - Material Deprivation and Economic Strain. Eurostat. 2023.

[33] Grol-Prokopczyk H, Freese J, Hauser RM. Using anchoring vignettes to assess group differences in general self-rated health. J Health Soc Behav. 2011;52(2):246–61. doi: 10.1177/0022146510396713 21673148 PMC3117438

[34] Stronks K, van de Mheen HD, Mackenbach JP. A higher prevalence of health problems in low income groups: Does it reflect relative deprivation?. J Epidemiol Community Health. 1998;52(9):548–57. doi: 10.1136/jech.52.9.548 10320855 PMC1756763

[35] Arber S, Fenn K, Meadows R. Subjective financial well-being, income and health inequalities in mid and later life in britain. Social Science & Medicine. 2014;100:12–20. 24444834 10.1016/j.socscimed.2013.10.016

[36] Landes SD, Ardelt M, Vaillant GE, Waldinger RJ. Childhood adversity, midlife generativity, and later life well-being. Journals of Gerontology Series B: Psychological Sciences and Social Sciences. 2014;69(6):942–52. 24870028 10.1093/geronb/gbu055 PMC6392441

[37] Gilbert N. European measures of poverty and “social exclusion”: Material deprivation, consumption, and life satisfaction. J Policy Anal Manage. 2009;28(4):738–44. doi: 10.1002/pam.20471

[38] Corna LM. A life course perspective on socioeconomic inequalities in health: A critical review of conceptual frameworks. Adv Life Course Res. 2013;18(2):150–9. doi: 10.1016/j.alcr.2013.01.002 24796266

[39] Doebler S, Glasgow N. Relationships between deprivation and the self-reported health of older people in Northern Ireland. J Aging Health. 2017;29(4):594–619. doi: 10.1177/0898264316641079 27034091

[40] Oldenburg B, Sallis JF, Harris D, Owen N. Checklist of Health Promotion Environments at Worksites (CHEW): Development and measurement characteristics. Am J Health Promot. 2002;16(5):288–99. doi: 10.4278/0890-1171-16.5.288 12053440

[41] Vignoli D, De Santis G. Individual and contextual correlates of economic difficulties in old age in Europe. Popul Res Policy Rev. 2009;29(4):481–501. doi: 10.1007/s11113-009-9153-6

[42] Li Y, Mutchler JE, Miller EA, Xiao JJ, Tucker-Seeley R. Space, context, and human capital: A micro–macro perspective on the social environment and financial literacy in later life. Popul Res Policy Rev. 2022;41(3):1385–404. doi: 10.1007/s11113-021-09695-y

[43] Godhwani S, Jivraj S, Marshall A, Bécares L. Comparing subjective and objective neighbourhood deprivation and their association with health over time among older adults in England. Health Place. 2019;55:51–8. doi: 10.1016/j.healthplace.2018.10.006 30472036

[44] Runciman WG. Relative deprivation and social justice: A study of attitudes to social inequality in twentieth-century England. 1966.

[45] Crosby F. A model of egoistical relative deprivation. Psychological Review. 1976;83(2):85–113. doi: 10.1037/0033-295x.83.2.85

[46] Smith HJ, Huo YJ. Relative deprivation: How subjective experiences of inequality influence social behavior and health. Policy insights from the behavioral and brain sciences. 2014;1(1):231–8.

[47] Oishi S, Kesebir S, Diener E. Income inequality and happiness. Psychol Sci. 2011;22(9):1095–100. doi: 10.1177/0956797611417262 21841151

[48] Winkleby M, Cubbin C, Ahn D. Effect of cross-level interaction between individual and neighborhood socioeconomic status on adult mortality rates. Am J Public Health. 2006;96(12):2145–53. doi: 10.2105/AJPH.2004.060970 17077398 PMC1698146

[49] Kessler RC, Duncan GJ, Gennetian LA, Katz LF, Kling JR, Sampson NA, et al. Associations of housing mobility interventions for children in high-poverty neighborhoods with subsequent mental disorders during adolescence. JAMA. 2014;311(9):937–48. doi: 10.1001/jama.2014.607 24595778 PMC4100467

[50] Smith HJ, Pettigrew TF, Pippin GM, Bialosiewicz S. Relative deprivation: A theoretical and meta-analytic review. Pers Soc Psychol Rev. 2012;16(3):203–32. doi: 10.1177/1088868311430825 22194251

[51] Lilly KJ, Sibley CG, Osborne D. Perceived relative deprivation across the adult lifespan: An examination of aging and cohort effects. Pers Soc Psychol Bull. 2025;51(4):554–72. doi: 10.1177/01461672231195332 37667668 PMC11827282

[52] Bardage C, Pluijm SMF, Pedersen NL, Deeg DJH, Jylhä M, Noale M, et al. Self-rated health among older adults: A cross-national comparison. Eur J Ageing. 2005;2(2):149–58. doi: 10.1007/s10433-005-0032-7 28794727 PMC5547684

[53] Guio AC, Marlier E. Alternative vs. current measures of material deprivation at EU level: what differences does it make?. 2013.

[54] Okulicz-Kozaryn A. Income and well-being across European provinces. Social Indicators Research. 2012;106(2):371–92.

[55] Helliwell JF, Huang H. New measures of the costs of unemployment: Evidence from the subjective well‐being of 3.3 million americans. Economic Inquiry. 2014;52(4):1485–502. doi: 10.1111/ecin.12093

[56] Ono H, Lee KS. Redistributing happiness: How social policies shape life satisfaction. Bloomsbury Publishing USA. 2016.

[57] European Commission. Strategic Foresight Report: Sustainability and people’s wellbeing at the heart of Europe’s Open Strategic Autonomy (COM(2023)376 final). Bruxelles: European Commission 2023.

[58] Navarro M, D’Agostino A, Neri L. The effect of urbanization on subjective well-being: Explaining cross-regional differences. Socio-Economic Planning Sciences. 2020;71:100824. doi: 10.1016/j.seps.2020.100824

[59] Chatterji M. Tertiary education and economic growth. Regional Studies. 1998;32(4):349–54. doi: 10.1080/00343409850117807

[60] Bertacchini E, Venturini A, Zotti R, Misuraca R. Life satisfaction and the variety and intensity of cultural participation in Italy: Disentangling the individual and regional dimensions. Regional Studies. 2024;59(1). doi: 10.1080/00343404.2024.2417701

[61] Carella M, Misuraca R. Subjective well being and heterogeneity in cultural consumption in the aging populations. Rivista Italiana di Economia, Demografia e Statistica. 2024;78(1):149–60.

[62] Carella M, Misuraca R. Gender differences and physical limitations in the association between subjective well-being and cultural consumption among older people. J Happiness Stud. 2025;26(3). doi: 10.1007/s10902-025-00875-7

[63] Armenise M, Benassi F, Carella M, Misuraca R. Accessibility and older and foreign populations: Exploring local spatial heterogeneities across Italy. Economies. 2024;12(9):248. doi: 10.3390/economies12090248

[64] Lewis JB, Linzer DA. Estimating regression models in which the dependent variable is based on estimates. Polit anal. 2005;13(4):345–64. doi: 10.1093/pan/mpi026

[65] Diez Roux AV. Investigating neighbourhood and area effects on health. American Journal of Public Health. 2001;91(11):1783–9. 11684601 10.2105/ajph.91.11.1783 PMC1446876

[66] Stafford M, Marmot M. Neighbourhood deprivation and health: does it affect us all equally?. Int J Epidemiol. 2003;32(3):357–66. doi: 10.1093/ije/dyg084 12777420

[67] Ager A, Pepper K. Patterns of health service utilization and perceptions of needs and services in rural Orissa. Health Policy and Planning. 2005;20(3):176–84. 15840633 10.1093/heapol/czi021

[68] Ross CE, Mirowsky J. Neighborhood disadvantage, disorder, and health. J Health Soc Behav. 2001;42(3):258–76. doi: 10.2307/3090214 11668773

