# Correction: Reconsidering the context in the relationship between material deprivation and self-rated health among older people in Italy

**DOI:** 10.1371/journal.pone.0350756

**Published:** 2026-06-01

**Authors:** Roberta Misuraca, Maria Carella

The ORCID iD is missing for the second author. Please see the authors’ respective ORCID iD here:

- Author Maria Carella’s ORCID iD is: 0000-0002-2116-8721 (https://orcid.org/0000-0002-2116-8721).

In the Author Contributions, Maria Carella should be listed in the category Writing – original draft.

## References

[1] Misuraca R, Carella M. Reconsidering the context in the relationship between material deprivation and self-rated health among older people in Italy. PLoS One. 2026;21(4):e0345858. doi: 10.1371/journal.pone.0345858 42008442 PMC13095002

