# Supplementary material for: Reconsidering the context in the relationship between material deprivation and self-rated health among older people in Italy
Source: PLoS One. 2026 Apr 20;21(4):e0345858. doi: 10.1371/journal.pone.0345858 (PMC13095002; doi:10.1371/journal.pone.0345858)
Supplement: S1 Appendix — This appendix reports additional descriptive statistics (Table A1 in S1 Appendix), second-step FGLS estimates for the population aged 55–64 (Table A2 in S1 Appendix), robustness checks using the continuous latent class posterior probability of material deprivation derived from the Latent Class Analysis (Table A3 in S1 Appendix), alternative model specifications treating self-rated health as an ordinal outcome (Table A4 in S1 Appendix), and multilevel logistic models with random intercepts and cross-level interactions between individual material deprivation and regional contextual factors (Table A5 in S1 Appendix). It also provides methodological details on the construction of the material deprivation indicator based on Latent Class Analysis. (DOCX) [file pone.0345858.s001.docx]

**Appendix**

Table A1. Descriptive statistics of the main variables

| Variable | Obs | Mean | Std. Dev. | Min | Max |
| --- | --- | --- | --- | --- | --- |
|  |  |  |  |  |  |
| SRH | 15,713 | 0.5722014 | 0.4947752 | 0 | 1 |
| DI | 15,713 | 0.2069624 | 0.4051412 | 0 | 1 |
|  |  |  |  |  |  |
| **Socio-demographics** |  |  |  |  |  |
| Gender |  |  |  |  |  |
| Male | 15,713 | 0.4559919 | 0.4980754 | 0 | 1 |
| Female | 15,713 | 0.5440081 | 0.4980754 | 0 | 1 |
|  |  |  |  |  |  |
| Age |  |  |  |  |  |
| 55-59 | 15,713 | 0.1981162 | 0.3985929 | 0 | 1 |
| 60-64 | 15,713 | 0.1745052 | 0.3795554 | 0 | 1 |
| 65-74 | 15,713 | 0.3127983 | 0.4636477 | 0 | 1 |
| >=75 | 15,713 | 0.3145803 | 0.4643633 | 0 | 1 |
|  |  |  |  |  |  |
| Marital status |  |  |  |  |  |
| Single | 15,602 | 0.0751827 | 0.2636943 | 0 | 1 |
| Married/Cohabitant | 15,602 | 0.6325471 | 0.4821267 | 0 | 1 |
| Separated/divorced | 15,602 | 0.0885143 | 0.2840505 | 0 | 1 |
| Widow/er | 15,602 | 0.2037559 | 0.4028025 | 0 | 1 |
| Education |  |  |  |  |  |
| PhD/degree | 15,598 | 0.0933453 | 0.290925 | 0 | 1 |
| Diploma | 15,598 | 0.2665726 | 0.4421812 | 0 | 1 |
| Secondary school | 15,598 | 0.2936915 | 0.4554669 | 0 | 1 |
| Primary school | 15,598 | 0.3463906 | 0.4758347 | 0 | 1 |
|  |  |  |  |  |  |
| Labour status |  |  |  |  |  |
| Occupied | 15,713 | 0.2206453 | 0.414695 | 0 | 1 |
| Job seeker | 15,713 | 0.0325208 | 0.1773844 | 0 | 1 |
| Unemployed | 15,713 | 0.7374785 | 0.4400185 | 0 | 1 |
| Not defined | 15,713 | 0.0093553 | 0.0962724 | 0 | 1 |
|  |  |  |  |  |  |
| Living arrangements |  |  |  |  |  |
| Living alone | 15,713 | 0.2568574 | 0.436914 | 0 | 1 |
| Couples | 15,713 | 0.6004582 | 0.4898198 | 0 | 1 |
| With family members | 15,713 | 0.0628779 | 0.2427509 | 0 | 1 |
| With distant relatives | 15,713 | 0.0463311 | 0.2102078 | 0 | 1 |
| housemates or caregivers | 15,713 | 0.0099917 | 0.0994612 | 0 | 1 |
|  |  |  |  |  |  |
| Income sources |  |  |  |  |  |
| Employment income | 15,475 | 0.1597415 | 0.366378 | 0 | 1 |
| Self-employment income | 15,475 | 0.0640388 | 0.2448299 | 0 | 1 |
| Pension | 15,475 | 0.6147981 | 0.4866587 | 0 | 1 |
| Allowance | 15,475 | 0.0264297 | 0.1604146 | 0 | 1 |
| Property income | 15,475 | 0.0094992 | 0.0970029 | 0 | 1 |
| Family maintenance | 15,475 | 0.1254927 | 0.3312875 | 0 | 1 |
|  |  |  |  |  |  |
| **Regional-level** |  |  |  |  |  |
| Unemployment rate | 15,713 | 11.07477 | 5.535452 | 3.804193 | 21.54494 |
| Per capita GDP | 15,713 | 0.028521 | 0.0080195 | 0.0168236 | 0.0433214 |
| CO2 emissions | 15,713 | 1.989002 | 0.465412 | 1.469702 | 4.106657 |
| Poverty index | 15,713 | 12.42592 | 7.752543 | 4.11 | 30.58 |
| Tertiary education rate | 15,713 | 27.80821 | 5.040636 | 20.33219 | 34.73656 |
| Number of beds in hospital facilities | 15,713 | 3.099025 | 0.3113715 | 2.54 | 3.68 |
| Elderly care facilities | 15,713 | 23.96905 | 11.26104 | 7.645233 | 54.49089 |
| Number of beds in care facilities | 15,713 | 739.2112 | 324.237 | 194 | 1343 |

Table A2. Regressions FGLS - Moderating effects of regional-level variables on the relationship between SRH and material deprivation

|  | |  | | |  | | (7) | (8) |
| --- | --- | --- | --- | --- | --- | --- | --- | --- |
| VARIABLES | (1) | (2) | (3) | (4) | (5) | (6) |  |  |
|  |  |  |  |  |  |  |  |  |
| Unemployment rate | -0.00616** |  |  |  |  |  |  |  |
|  | (0.00277) |  |  |  |  |  |  |  |
| Per capita GDP |  | 4.937** |  |  |  |  |  |  |
|  |  | (1.743) |  |  |  |  |  |  |
| CO2 emissions |  |  | 0.0811*** |  |  |  |  |  |
|  |  |  | (0.0198) |  |  |  |  |  |
| Poverty index |  |  |  | -0.00485** |  |  |  |  |
|  |  |  |  | (0.00184) |  |  |  |  |
| Tertiary education rate |  |  |  |  | 0.00643** |  |  |  |
|  |  |  |  |  | (0.00306) |  |  |  |
| Number of beds in hospital facilities |  |  |  |  |  | 0.157*** |  |  |
|  |  |  |  |  |  | (0.0384) |  |  |
| Elderly care facilities |  |  |  |  |  |  | 0.00445*** |  |
|  |  |  |  |  |  |  | (0.000988) |  |
| Number of beds in care facilities |  |  |  |  |  |  |  | 0.000142*** |
|  |  |  |  |  |  |  |  | (3.97e-05) |
| Constant | 0.143*** | -0.0632 | -0.0930** | 0.137*** | -0.101 | -0.414*** | -0.0374 | -0.0311 |
|  | (0.0337) | (0.0509) | (0.0429) | (0.0269) | (0.0854) | (0.120) | (0.0275) | (0.0323) |
|  |  |  |  |  |  |  |  |  |
| Observations | 20 | 20 | 20 | 20 | 20 | 20 | 20 | 20 |
| R-squared | 0.216 | 0.308 | 0.482 | 0.280 | 0.197 | 0.482 | 0.530 | 0.416 |

Standard errors in parentes; *** p<0.01, ** p<0.05, * p<0.010. The reference population are individuals aged 55-64. Each column (1 to 8) represents a separate regression model, including one regional-level variable at a time. The dependent variable is ($\hat{\beta}$), the estimated effect of *DI* on *SRH.*

**Latent class analysis approach**

To understand how the combined influence of the variety and frequency of deprivation indicators is related to SHR, we employed Carella and Misuraca’s (2025) latent class analysis (LCA) approach, from which we derived a measure of individual high material deprivation:

**
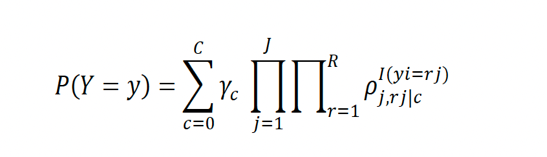
**

Where $P(Y = y)$ denotes the likelihood of observing a set of responses given $I(y_{j}=r_{j})$ if the response to variable $j= rj$ or 0 otherwise; $\gamma_{c}$ represents the likelihood of an individual belonging to latent class *c*, while $\rho_{j,rj|c)}^{I(y_{i} = r_{j})}$is the probability of response $r_{j}$ for each individual *i.* The key parameters in the model are 𝛾, indicating the probability of membership in a given latent class, and *ρ*, which represents the conditional probabilities of item responses given class membership 𝛾.

This latent class analysis enabled us to assign each participant to the class that most accurately reflected their specific characteristics. These classes were mutually exclusive, meaning each individual was classified into only one. By segmenting the sample into well-defined subgroups, we were able to conduct a detailed analysis. The fundamental principle of LCA is to construct categories consisting of individuals with similar response patterns. In this case, we grouped individuals based on shared material deprivation patterns, considering both the breadth and severity of various deprivation indicators.

Table A3. Regressions FGLS - Moderating effects of regional-level variables on the relationship between SRH and high material deprivation profile obtained through the LCA approach.

|  |  |  |  |  |  |  |  |  |
| --- | --- | --- | --- | --- | --- | --- | --- | --- |
| VARIABLES | (1) | (2) | (3) | (4) | (5) | (6) | (7) | (8) |
|  |  |  |  |  |  |  |  |  |
| Unemployment rate | -0.0104** |  |  |  |  |  |  |  |
|  | (0.00428) |  |  |  |  |  |  |  |
| Per capita GDP |  | 8.528*** |  |  |  |  |  |  |
|  |  | (2.631) |  |  |  |  |  |  |
| CO2 emissions |  |  | 0.147*** |  |  |  |  |  |
|  |  |  | (0.0263) |  |  |  |  |  |
| Poverty index |  |  |  | -0.00765** |  |  |  |  |
|  |  |  |  | (0.00290) |  |  |  |  |
| Tertiary education rate |  |  |  |  | 0.00980 |  |  |  |
|  |  |  |  |  | (0.00487) |  |  |  |
| Number of beds in hospital facilities |  |  |  |  |  | 0.246*** |  |  |
|  |  |  |  |  |  | (0.0612) |  |  |
| Elderly care facilities |  |  |  |  |  |  | 0.00786*** |  |
|  |  |  |  |  |  |  | (0.00132) |  |
| Number of beds in care facilities |  |  |  |  |  |  |  | 0.000233*** |
|  |  |  |  |  |  |  |  | (6.10e-05) |
| Constant | 0.229*** | -0.126 | -0.191*** | 0.210*** | -0.156 | -0.651*** | -0.0852** | -0.0605 |
|  | (0.0522) | (0.0768) | (0.0569) | (0.0425) | (0.136) | (0.191) | (0.0368) | (0.0496) |
|  |  |  |  |  |  |  |  |  |
| Observations | 20 | 20 | 20 | 20 | 20 | 20 | 20 | 20 |
| R-squared | 0.246 | 0.368 | 0.634 | 0.279 | 0.184 | 0.473 | 0.662 | 0.447 |

Standard errors in parentheses; *** p<0.01, ** p<0.05, * p<0.010. The reference population are individuals aged 65 and older. Each column (1 to 8) represents a separate regression model, including one regional-level variable at a time. The dependent variable is ($\hat{\beta}$), the estimated effect of *DI* on *SRH.* The main explanatory variable is a material deprivation profile obtained through the implementation of LCA and captures different conditional marginal probabilities of being materially deprived at the individual level.

Table A4. Regressions FGLS - Moderating effects of regional-level variables on the relationship between ordinal SRH variable and high material deprivation.

| VARIABLES | (1) | (2) | (3) | (4) | (5) | (6) | (7) | (8) |
| --- | --- | --- | --- | --- | --- | --- | --- | --- |
|  |  |  |  |  |  |  |  |  |
| Unemployment rate | -0.0112** |  |  |  |  |  |  |  |
|  | (0.00479) |  |  |  |  |  |  |  |
| Per capita GDP |  | 8.282** |  |  |  |  |  |  |
|  |  | (3.116) |  |  |  |  |  |  |
| CO2 Emissions |  |  | 0.113** |  |  |  |  |  |
|  |  |  | (0.0402) |  |  |  |  |  |
| Poverty Index |  |  |  | -0.00609 |  |  |  |  |
|  |  |  |  | (0.00351) |  |  |  |  |
| Tertiary education rate |  |  |  |  | 0.0123** |  |  |  |
|  |  |  |  |  | (0.00523) |  |  |  |
| Number of beds in hospital facilities |  |  |  |  |  | 0.262*** |  |  |
|  |  |  |  |  |  | (0.0705) |  |  |
| Elderly care facilities |  |  |  |  |  |  | 0.00821*** |  |
|  |  |  |  |  |  |  | (0.00163) |  |
| Number of beds in care facilities |  |  |  |  |  |  |  | 0.000270*** |
|  |  |  |  |  |  |  |  | (6.51e-05) |
| Constant | 0.301*** | -0.0556 | -0.0580 | 0.254*** | -0.162 | -0.636*** | -0.0310 | -0.0254 |
|  | (0.0584) | (0.0909) | (0.0871) | (0.0514) | (0.146) | (0.220) | (0.0452) | (0.0530) |
|  |  |  |  |  |  |  |  |  |
| Observations | 20 | 20 | 20 | 20 | 20 | 20 | 20 | 20 |
| R-squared | 0.234 | 0.282 | 0.305 | 0.143 | 0.235 | 0.433 | 0.586 | 0.489 |

Standard errors in parentheses; *** p<0.01, ** p<0.05, * p<0.010. The reference population are individuals aged 65 and older. Each column (1 to 8) represents a separate regression model, including one regional-level variable at a time. The dependent variable is ($\hat{\beta}{DI}_{i,r}$), the estimated effect of *DI* on *SRH,* an ordinal variable evaluated using a 5-point Likert scale, from 1 (indicating very good) to 5 (indicating very bad) in the past 12 months*.*

Table A5. Multilevel Logistic Models with Cross-Level Interactions between Individual Material Deprivation and Regional Contextual Factors

| VARIABLES | | | | (1) | | (2) | | (3) | | (4) | | (5) | (6) | (7) | (8) |
| --- | --- | --- | --- | --- | --- | --- | --- | --- | --- | --- | --- | --- | --- | --- | --- |
|  |  |  |  | |  | |  | |  | |  |  |  |  |  |
|  | | | |  | |  | |  | |  | |  |  |  |  |
| High material deprivation Index | | | | 0.383*** | | 0.389*** | | 0.399*** | | 0.386*** | | 0.390*** | 0.404*** | 0.405*** | 0.398*** |
|  | | | | (0.0601) | | (0.0597) | | (0.0599) | | (0.0598) | | (0.0600) | (0.0603) | (0.0600) | (0.0599) |
| Unemployment rate | | | | 0.367*** | |  | |  | |  | |  |  |  |  |
|  | | | | (0.0509) | |  | |  | |  | |  |  |  |  |
| Highmaterdepriv*Unemploy rate | | | | -0.128** | |  | |  | |  | |  |  |  |  |
|  | | | | (0.0558) | |  | |  | |  | |  |  |  |  |
| Per capita GDP | | | |  | | -0.342*** | |  | |  | |  |  |  |  |
|  | | | |  | | (0.0545) | |  | |  | |  |  |  |  |
| Highmaterdepriv*Per capita GDP | | | |  | | 0.125** | |  | |  | |  |  |  |  |
|  | | | |  | | (0.0570) | |  | |  | |  |  |  |  |
| CO2 emissions | | | |  | |  | | -0.244*** | |  | |  |  |  |  |
|  | | | |  | |  | | (0.0533) | |  | |  |  |  |  |
| Highmaterdepriv*CO2 emissions | | | |  | |  | | 0.182*** | |  | |  |  |  |  |
|  | | | |  | |  | | (0.0593) | |  | |  |  |  |  |
| Poverty index | | | |  | |  | |  | | 0.340*** | |  |  |  |  |
|  | | | |  | |  | |  | | (0.0570) | |  |  |  |  |
| Highmaterdepriv*Poverty index | | | |  | |  | |  | | -0.125** | |  |  |  |  |
|  | | | |  | |  | |  | | (0.0554) | |  |  |  |  |
| Tertiary education rate | | | |  | |  | |  | |  | | -0.308*** |  |  |  |
|  | | | |  | |  | |  | |  | | (0.0650) |  |  |  |
| Highmaterdepriv*Tertiary education rate | | | |  | |  | |  | |  | | 0.124** |  |  |  |
|  | | | |  | |  | |  | |  | | (0.0570) |  |  |  |
| Number of beds in hospital facilities | | | |  | |  | |  | |  | |  | -0.311*** |  |  |
|  | | | |  | |  | |  | |  | |  | (0.0672) |  |  |
| Highmaterdepriv*Number of beds in hospital facilities | | | |  | |  | |  | |  | |  | 0.215*** |  |  |
|  | | | |  | |  | |  | |  | |  | (0.0595) |  |  |
| Elderly care facilities | | | |  | |  | |  | |  | |  |  | -0.283*** |  |
|  | | | |  | |  | |  | |  | |  |  | (0.0669) |  |
| Highmaterdepriv*Elderly care facilities | | | |  | |  | |  | |  | |  |  | 0.250*** |  |
|  | | | |  | |  | |  | |  | |  |  | (0.0617) |  |
| Number of beds in care facilities | | | |  | |  | |  | |  | |  |  |  | -0.331*** |
|  | | | |  | |  | |  | |  | |  |  |  | (0.0598) |
| Highmaterdepriv*Number of beds in care facilities | | | |  | |  | |  | |  | |  |  |  | 0.202*** |
|  | | | |  | |  | |  | |  | |  |  |  | (0.0565) |
| Constant | | | | -1.510*** | | -1.505*** | | -1.470*** | | -1.505*** | | -1.511*** | -1.495*** | -1.477*** | -1.494*** |
|  | | | | (0.210) | | (0.211) | | (0.214) | | (0.212) | | (0.214) | (0.215) | (0.215) | (0.212) |
| Constant by region | | | | 0.0315** | | 0.0417** | | 0.0685*** | | 0.0455** | | 0.0630*** | 0.0722*** | 0.0768*** | 0.0539** |
|  | | | | (0.0136) | | (0.0166) | | (0.0262) | | (0.0178) | | (0.0236) | (0.0261) | (0.0283) | (0.0210) |
|  | | | |  | |  | |  | |  | |  |  |  |  |
| Observations | | | | 9,651 | | 9,651 | | 9,651 | | 9,651 | | 9,651 | 9,651 | 9,651 | 9,651 |
| Number of groups | | | | 20 | | 20 | | 20 | | 20 | | 20 | 20 | 20 | 20 |

Notes: Estimates are adjusted for gender; age; partnership status (married/cohabiting, separated/divorced, widowed, or single); educational attainment (upper-secondary or tertiary education versus low education); living arrangements (living alone, with family members, or with distant relatives/housemates/caregivers); labour status (employed, retired, or unemployed); and income sources. The analytical sample includes individuals aged 65 and older. All regional-level predictors were standardized. Standard errors are clustered at the regional level through random-intercept specification. *** p<0.01, ** p<0.05, * p<0.010.
